# Stroma AReactive Invasion Front Areas (SARIFA): a novel histopathologic biomarker in colorectal cancer patients and its association with the luminal tumour proportion

**DOI:** 10.1016/j.tranon.2024.101913

**Published:** 2024-04-10

**Authors:** N.G. Reitsam, B. Grosser, J.S. Enke, W. Mueller, A. Westwood, N.P. West, P. Quirke, B. Märkl, H.I. Grabsch

**Affiliations:** a Pathology, Faculty of Medicine, University of Augsburg, Augsburg, Germany; b Nuclear Medicine, Faculty of Medicine, University of Augsburg, Augsburg, Germany; c Gemeinschaftspraxis Pathologie, Starnberg, Germany; d Division of Pathology and Data Analytics, Leeds Institute of Medical Research at St James's University, University of Leeds, Leeds, UK; e Department of Pathology, GROW - Research Institute for Oncology and Reproduction, Maastricht University Medical Center+, Maastricht, The Netherlands

**Keywords:** Colorectal cancer, Biomarker, Tumour stroma, Rectal cancer, Gastrointestinal oncology

## Abstract

**Background:**

Stroma AReactive Invasion Front Areas (SARIFA) is a novel prognostic histopathologic biomarker measured at the invasive front in haematoxylin & eosin (H&E) stained colon and gastric cancer resection specimens. The aim of the current study was to validate the prognostic relevance of SARIFA-status in colorectal cancer (CRC) patients and investigate its association with the luminal proportion of tumour (PoT).

**Methods:**

We established the SARIFA-status in 164 CRC resection specimens. The relationship between SARIFA-status, clinicopathological characteristics, recurrence-free survival (RFS), cancer-specific survival (CSS), and PoT was investigated.

**Results:**

SARIFA-status was positive in 22.6% of all CRCs. SARIFA-positivity was related to higher pT, pN, pTNM stage and high grade of differentiation. SARIFA-positivity was associated with shorter RFS independent of known prognostic factors analysing all CRCs (RFS: hazard ratio (HR) 2.6, *p* = 0.032, CSS: HR 2.4, *p* = 0.05) and shorter RFS and CSS analysing only rectal cancers. SARIFA-positivity, which was measured at the invasive front, was associated with PoT-low (*p* = 0.009), e.g., higher stroma content, and lower vessel density (*p* = 0.0059) measured at the luminal tumour surface.

**Conclusion:**

Here, we validated the relationship between SARIFA-status and prognosis in CRC patients and provided first evidence for a potential prognostic relevance in the subgroup of rectal cancer patients. Interestingly, CRCs with different SARIFA-status also showed histological differences measurable at the luminal tumour surface. Further studies to better understand the relationship between high luminal intratumoural stroma content and absence of a stroma reaction at the invasive front (SARIFA-positivity) are warranted and may inform future treatment decisions in CRC patients.

## Highlights of the current study

• SARIFA-status proves to be a prognostic H&E based biomarker in colorectal cancer.
• SARIFA-positivity is strongly associated with known high-risk features.
• SARIFA-positive CRC patients show poor survival outcomes.
• There are histological differences at the luminal tumour component in SARIFA-positive CRCs.
• SARIFA-positivity is associated with a lower proportion of tumour (higher stroma content).

## Introduction

Colorectal cancer (CRC) is the third most common cancer worldwide, contributing significantly to the global burden of disease [1]. Radiological and/or pathological disease stage according to AJCC/UICC tumour node metastasis (TNM) [2] is currently the most important tool for therapeutic decision-making in CRC patients. However, TNM stage, and other histological biomarkers such as grade of differentiation and tumour budding [3], are unable to predict survival for individual patients resulting in potential under- or overtreatment of some patients [4]. Molecular tumour characteristics such as DNA mismatch repair status (MMR status) are becoming increasingly important, especially for the treatment of patients with distant metastases [5] or when considering immunotherapy [6]. RNA expression-based approaches to classify CRC into different potentially clinically relevant subtypes such as the consensus molecular subtypes (CMS) [7] or CINSARC [8] have been proposed. However, this approach requires technically challenging assays which are expensive and difficult to introduce into daily clinical routine. Therefore, there remains an urgent need for new robust, cheap and easy-to-implement biomarkers in CRC.

There have been several studies highlighting the relevance of tumour-stroma ratio/tumour cell proportion/tumour cell density/proportion of tumour (PoT) for prognosis prediction in CRC patients: High intra-tumour stroma content measured using different methods has been shown to predict poor prognosis in several different CRC patient cohorts [9], [10], [11], [12], [13], [14], [15] and other tumour types. Moreover, it has been suggested that tumour stroma is actively involved in CRC development and progression, and that disruption of the tumour-stroma interaction may inhibit tumour progression and metastasis formation [16].

Our group has been focussing on studying the stroma-tumour interactions at the deep invasion front, and we were the first to identify the presence of **S**troma **AR**eactive **I**nvasion **F**ront **A**reas (SARIFA) as a new Haematoxylin & Eosin (H&E) based prognostic biomarker in patients with colon or gastric cancer [[17], [18]]. SARIFA-positivity is defined as the direct contact between tumour cells and adipocytes at the invasion front without intervening stroma (e.g. without the usually seen so-called desmoplastic stroma reaction) or intervening inflammatory infiltrate, i.e. absence of lymphocytes, plasma cells and granulocytes. We demonstrated previously that SARIFA-status can be determined with high interobserver agreement in a timely fashion [[17], [18]], using routine H&E slides. Moreover, we showed in gastric cancer that SARIFA-positivity is associated with an upregulation of lipid metabolism in tumour cells [17] and an altered immune response, especially a substantial decrease in antitumoural natural killers cell in the peripheral blood of patients with SARIFA-positive CRCs [19], suggesting distinct tumour biology behind SARIFA. Targeting these particular biological properties could provide new treatment opportunities for patients with SARIFA-positive CRCs.

Although SARIFA-positivity is histologically only visible at the tumour fat interface, SARIFA-positivity might be related to other features related to a poor prognosis and measurable in the tumour centre or at the luminal (e.g., endoscopically reachable) tumour surface. As both, SARIFA-positivity and high intratumoural stroma content, are negative prognostic factors related to the tumour microenvironment, we hypothesised that SARIFA-positivity may be related to high intratumour stroma content (low PoT), a histological biomarker with prognostic value in colorectal cancer, which is – in contrast to SARIFA-status – measurable at the luminal tumour surface [14].

The aims of the current study were (1) to validate the prognostic relevance of the SARIFA-status in a series of patients with colorectal cancers independent of our initial series [18], (2) to explore the prognostic value of the SARIFA-status in the subgroup of patients with rectal cancer and (3) to further characterise SARIFA-positive colorectal cancer to better understand the underlying tumour biology, in particular by investigating the intratumour stroma content (PoT).

## Material & methods

### Patients

Patients who had undergone potentially curative resection for colorectal cancer (CRC) at the Marienhospital, Düsseldorf, Germany, between January 1990 and December 1995 were included in this retrospective study of consecutive cases without matching or randomization.

**Fig. 1:**
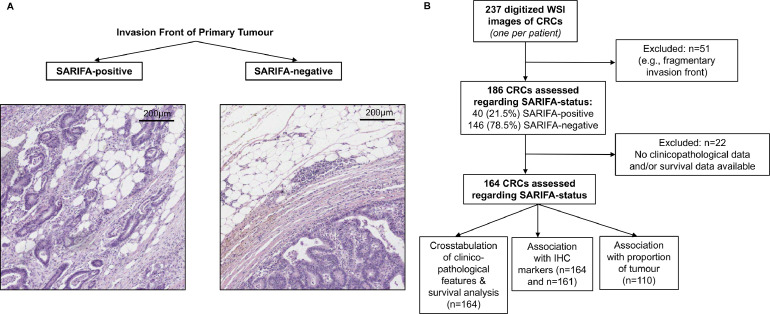
Definition of SARIFA and study design. A. Left panel: Haematoxylin & Eosin stained tissue section of colorectal cancer classified as SARIFA-positive, as tumour cells at the invasion front are seen directly adjacent to adipocytes at the invasion front. Right panel: SARIFA-negative colorectal cancer case with desmoplasia and/or immune infiltrate between tumour cells and adipocytes at the invasion front. B. Study flow chart: 237 digitized slides were initially screened; 186 were classifiable regarding SARIFA-status, of which 164 CRCs were included in the final analysis regarding relationship with clinicopathological features, survival analysis and IHC markers (*n* = 161). For a subset of patients, proportion of tumour data from an earlier study [14] was available. Overlap of CRC patients with SARIFA-status, PoT, and clinicopathological and survival data was *n* = 110. CRC: colorectal cancer, IHC: immunohistochemistry, SARIFA: Stroma AReactive Invasion Front Area, WSI: whole slide image.

Patient who had received pre-operative chemo- or radiotherapy were excluded. The median (range) age of all patients was 69.3 years (44.9 to 88.3 years). The follow-up time of SARIFA-positive and SARIFA-negative CRC patients was similar (median range, SARIFA-positive: 6.0 years [2.7–9.3 years]) vs SARIFA-negative: 4.8 years (4.3–5.2 years), *p* = 0.389). From all resection specimens, a single representative tumour containing H&E stained tissue section was scanned at 40x magnification (Aperio XT whole slide scanner, Aperio Technologies, Vista, CA, USA). H&E and immunohistochemically stained slides from this cohort have also been used in previous studies [[14], [20], [21]]. After screening digitized slides from 237 resection specimens, 186 CRC cases were deemed assessable for SARIFA-status (criteria used see below). Clinicopathological data were available for 164 CRC cases (Fig. 1). The Tumour-Node-Metastasis (TNM) classification used at the time of surgery was TNM, 5th edition (Sobin and Wittekind, 1997, pp 66–69) [22]. Since the 5th edition, the definition of pT and pN has not changed substantially [23]. Changes in TNM stage groupings in CRC mainly affected the evaluation of tumour deposits, which occur only in a comparably small subset of CRCs (around 20%) [24].

Primary study endpoints were cancer-specific survival (event = death due to cancer), and recurrence-free survival (event = death to cancer or local/distant disease recurrence). The study was performed in accordance with local ethics regulation, the need for patient consent was waived by the ethics committee.

In 51 (21.5%) of 237 whole slide images (WSI) of CRC resection slides, SARIFA-status could not be assessed reliably. The main reason for this was in particular a fragmentary invasion front; the tumour-fat interface is necessary for a reliable assessment of the SARIFA-status. Twenty-two CRC cases with SARIFA-status were excluded from further analysis because of missing clinicopathological or survival data. Thus, 164 CRC were included in the final analyses (Fig. 1).

This study conforms to the REMARK [25] guidelines (refer to Table S1), just like our initial internal biomarker discovery study on SARIFA in colon cancer [18].

### SARIFA assessment

SARIFA-positivity was defined as published in our previous studies in colon cancer/CRC [18,19]: the presence of an area within the deep tumour invasion front where at least a single tumour gland or group of ≥ 5 tumour cells are located directly adjacent to adipocytes without intervening stromal reaction or inflammatory infiltrate (Fig. 1). As soon as one area was classified as SARIFA-positivity, the whole case was classified as SARIFA-positive irrespective whether there were areas at the invasion front which were SARIFA-negative. If no SARIFA-positivity was present anywhere in the tissue section, the case was classified as SARIFA-negative. All cases were classified by NGR as SARIFA-positive, SARIFA-negative or non-assessable, a pathology trainee, who also assessed SARIFA-status in one of our previous studies on CRCs [19]. NGR was blinded to any clinicopathological data including outcome data. We have already shown that interobserver variability of SARIFA scoring is low [18]. SARIFA-status was assessed on H&E stained sections, scanned at 40x magnification and reviewed digitally. One single section (considered as most representative) of the primary tumour was assessed.

### Proportion of Tumour measurements

The luminal proportion of tumour (PoT) was morphometrically measured in a previous study by a so-called point counting approach. Details as well as visualisation of this technique can be found in the methods section of our previous publication [14]. In short, a 9 mm^2^ area with highest tumour cell density by eyeballing was selected at the luminal surface. A systematic random sample of 300 equally spaced points was used to quantify the relative proportion of the different tissue categories in the measurement area. Regions with ulcerations, extensive necrosis and presence of surface mucus were avoided when choosing the area of interest. The following categories were used for analyses in the current study: tumour, stroma, tumour lumen, necrosis, vessel, and inflammation. The dichotomisation of the PoT as high versus low was the same as in the previous study ^14^: PoT-high was defined as >47% of the area consists of tumour cells versus PoT-low with ≤47%.

### Ki67 and MLH1/MSH2 immunohistochemistry

To further analysis the underlying tumour biology, immunohistochemical data for Ki67 (proliferation marker) and MLH1/MSH2 (DNA mismatch repair status) from a previous study were used to explore potential associations with SARIFA-status. Details about the immunohistochemical procedures which were performed on tissue microarrays (TMAs) can be found in our previous study [20]. Tumours with complete nuclear loss of MLH1 or MSH2 expression, in the presence of internal positive controls, were considered MMR deficient (dMMR), whereas tumours that expressed both MMR proteins were considered MMR proficient (pMMR), as also described previously [23]. Cut offs for Ki67 low (<10% Ki67 positive tumour cells), moderate (>10% and <25%) and high (>25%) were used as previously published by Melling et al. [26].

### Statistical analyses

For hypothesis testing of differences between relative frequencies of categorial variables Chi-squared or Fisher's exact tests were used. Continuous variables were compared using the Mann-Whitney-*U test*. Estimates of Kaplan-Meier survival probabilities were compared using log-rank tests. The median follow up was calculated using the reverse Kaplan-Meier method [27]. Relative risks were estimated by hazard ratios (HRs), obtained by Cox proportional hazard models. In multivariate Cox regression analysis, the following variables (all known risk factors) were included in the model: pT category, pN category, grade of differentiation, PoT and SARIFA-status. We tested for the proportional hazards assumption of our Cox proportional hazards regression model using the ‘cox.zph’ function of the *survival* package. We tested for multicollinearity using the ‘vif’ function of the *car* package, and added the variance inflation factors (VIF). The results of these tests can be found in Table S2, and indicate that our model meets the assumption of proportional hazards. Furthermore, multicollinearity does not seem to be a limitation here. For statistical analyses, IBM SPSS Statistics (Version 29.0.0.0, IBM, Armonk, NY, USA) and R, version 4.2.2 (R Foundation for Statistical Computing, Vienne, Austria) were used. P-values < 0.05 were considered statistically significant. R packages used were *survival* (version 3.5–7), *survminer* (version 0.4.9), *car* (version 3.1–2), *ggplot2* (version 3.4.4), *ggpubr* (version 0.6.0), *ggsci* (version 3.0.0), *ggrepel* (version 0.9.4), and *dplyr* (version 1.1.4).

## Results

### SARIFA-status is associated with high-risk features in colorectal cancer

77.4% (*n* = 127) colorectal cancer (CRC) cases were classified as SARIFA-negative, 22.6% (*n* = 37) as SARIFA-positive. In the subgroup of patients with rectal cancers, 29.7% (11 out of 60) rectal cancers were classified as SARIFA-positive. SARIFA-positive CRCs had more frequently a higher pT category, a higher pN category, a higher TNM stage and were more frequently poorly differentiated (all p-values <0.001). Only one of the 49 pT1/pT2 CRCs was classified as SARIFA-positive as tumour cells were seen next to submucosally located adipocytes. Because of the higher TNM stage, patients with SARIFA-positive CRCs were more likely to be treated with adjuvant chemotherapy (frequency of adjuvant chemotherapy, SARIFA-positive: 27% versus SARIFA-negative: 9.4%, *p* = 0.006). Furthermore, there was a relationship between SARIFA-status measured at the invasion front and proportion of tumour measured at the luminal tumour surface: SARIFA-positive CRCs were more often found to be luminal PoT-low (e.g. stroma proportion high) (*p* = 0.009). There was no relationship between SARIFA-status and age, sex, tumour location (colon vs rectum) or lymphovascular invasion status (all p-values >0.05). There was no SARIFA-status related difference in age (median age [range] in years; all patients: 69.3 [44.9–88.3], SARIFA-positive patients: 69.35 [44.9–86.0], SARIFA-negative patients: 69.25 (47.5–88.3], *p* = 0.612). For a detailed overview of the clinicopathological characteristics of patients with SARIFA-positive and SARIFA-negative CRC, see Table 1.

**Table 1:** Relationship between Stroma AReactive Invasion Front Areas (SARIFA) and clinicopathological characteristics.

		All colorectal cancer		SARIFA-positive CRC		SARIFA-negative CRC		
		n	%	n	%	n	%	*p-*value
		164	100	37	22.6	127	77.4	
Sex								
	Male	73	43.3	20	54.1	51	40.2	0.133
	Female	93	56.7	17	45.9	76	59.8	
Tumour location								
	Colon	104	63.4	26	70.3	78	61.4	0.325
	Rectum	60	36.6	11	29.7	49	38.6	
pT category								
	pT1/pT2	49	29.9	1	2.7	48	37.8	**<0.001**
	pT3/pT4	115	70.1	36	97.3	79	62.2	
pN category								
	pN0	105	64.0	13	35.1	92	72.4	**<0.001**
	pN1	37	22.6	12	32.4	25	19.7	
	pN2	22	13.4	12	32.4	10	7.9	
TNM* stage								
	I/II	104	63.4	13	35.1	91	71.7	**<0.001**
	III/IV	60	36.6	24	64.9	36	28.3	
Adjuvant treatment								
	No	142	86.6	27	73.0	115	90.6	**0.006**
	Yes	22	13.4	10	27.0	12	9.4	
Lymphovascular invasion								
	No	115	70.1	24	64.9	91	71.1	0.427
	Yes	49	29.9	13	35.1	35	28.3	
Grade of differentiation								
	Low	125	76.2	19	51.4	106	83.5	**<0.001**
	High	39	23.8	18	48.6	21	16.5	
Proportion of tumour#								
	Low	26	23.6	12	41.4	14	17.3	**0.009**
	High	84	76.4	17	58.6	67	82.7	

*p*-values that are statistically significant are highlighted in **bold.**

⁎ Tumour-Node-Metastasis stage grouping was obtained using TNM, 5th edition (Sobin and Wittekind, 1997, pp 66–69) [22].

# PoT data were available for 110 patients only pT: depth of invasion, pN: lymph node status.

### Exploratory analysis of the relationship between luminal tumour composition and SARIFA-status

**Fig. 2:**
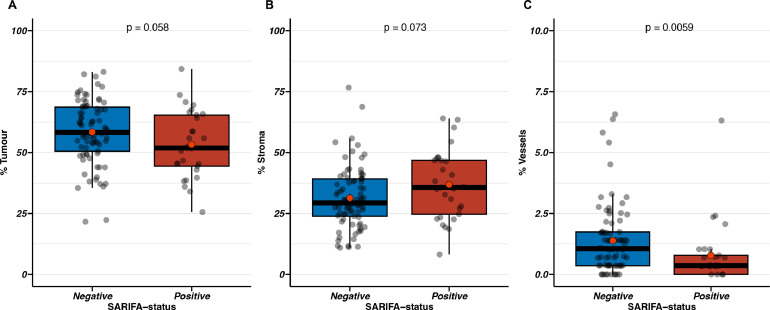
Bar charts illustrating the relationship between luminal tumour components and SARIFA-status (*n* = 110). A. SARIFA-negative CRCs show a trend towards higher tumour percentage (median (range), proportion of tumour in%: SARIFA-positive 51.86 (25.58–84.30) versus SARIFA-negative 59.46 (21.62–83.10), *p* = 0.058), B. SARIFA-positive CRCs show a trend towards higher stroma percentage (median (range), proportion of stroma in%: SARIFA-positive 35.69 (8.19–64.01) versus SARIFA-negative 28.97 (10.95–76.69), *p* = 0.073), C. SARIFA-positive CRCs show decreased vessel density (median (range), area covered by vessels in%: SARIFA-positive 0.36 (0.00–6.32) versus SARIFA-negative 1.07 (0.00–6.57). x-Axis: SARIFA-status (A-C), y-Axis: Percentage of luminal tumour component, 0–100% (A, B), 0–10% (C). Boxplots depict the interquartile range (IQR, Q1-Q3) with the median (Q2) also highlighted. The whiskers extend from the edges of the box to the minimum and maximum values within 1.5 x IQR; any data points beyond the whiskers are considered as outliers and shown as individual data points. SARIFA: Stroma AReactive Invasion Front Area.

To address whether SARIFA-status defined as ‘no stroma reaction’ and measured at the invasion front is associated with distinct histological findings measurable at the luminal surface, we investigated the relationship between SARIFA-status and previously measured proportion of tumour (PoT) and proportion of other components (stroma, inflammation, necrosis, vessels, and tumour lumen) at the luminal surface in the same slide [14]. PoT and related data were available for 110 CRC cases (67.07% of the entire cohort). Hereof, 81 (73.6%) were classified as SARIFA-negative and 29 (26.4%) as SARIFA-positive. Interestingly, SARIFA-positive CRCs seem to have lower tumour cell and higher stroma density at the luminal surface, although statistical significance was not reached, which is most likely related to the relatively small cohort size (*p* = 0.058 and *p* = 0.073, respectively, Fig. 2A, B). This is in line with the finding that SARIFA-positive CRCs more often belong to the PoT-low category [14] (see Table 1, PoT-low category: SARIFA-positive 41.4% versus SARIFA-negative 17.3%). Additionally, SARIFA-positive CRCs were characterized by a lower luminal proportion of vessels (*p* = 0.0059, Fig. 2C). No differences were observed regarding percentage of tumour lumen, necrosis, or inflammation (all *p* > 0.05).

### SARIFA-status as independent prognostic biomarker in patients with colorectal cancer

**Fig. 3:**
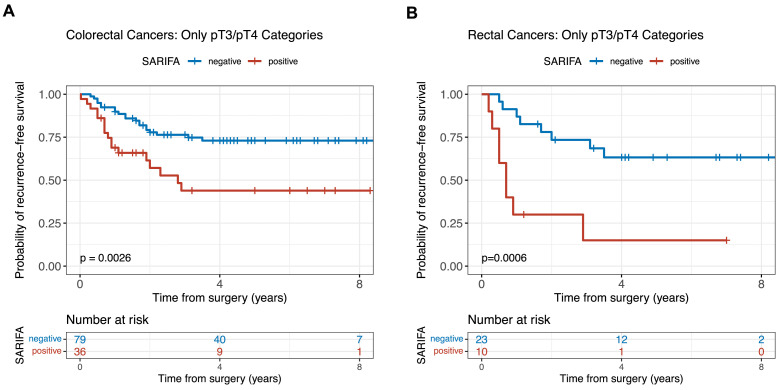
Kaplan-Meier curves for recurrence-free survival (RFS) stratified by SARIFA-status. A. Patients with locally advanced (pT3/pT4) SARIFA-positive colorectal cancer (*n* = 36) have a significantly shorter RFS (HR 2.63, 95% CI: 1.371–5.046, *p* = 0.0026). B. Patients with locally advanced (pT3/pT4) SARIFA-positive rectal cancer (*n* = 10) have a significantly shorter RFS (HR 5.143, 95% CI: 1.859–14.24, *p* = 0.0006). HR: hazard ratio. CI: confidence interval. SARIFA: Stroma AReactive Invasion Front Area.

We used univariate Kaplan-Meier analyses to establish the relationship between SARIFA-status and recurrence-free survival (RFS) or cancer-specific survival (CSS). There was a significant difference in RFS comparing SARIFA-positive and SARIFA-negative CRC (median RFS: SARIFA-negative CRC not reached, SARIFA-positive CRC 2.9 years; SARIFA-positive CRC 5-year RFS 46% versus 80% in patients with SARIFA-negative CRC; HR: 3.665, 95% CI: 1.949–6.890, *p* < 0.001.). As SARIFA-positive CRCs were almost exclusively locally advanced (see Table 1), we explored RFS in the subgroup of patients with pT3/pT4 CRCs. Patients with pT3/pT4 SARIFA-positive CRC also had a significantly shorter RFS compared to patients with SARIFA-negative pT3/pT4 CRCs (*p* = 0.0026). Furthermore, we explored the relationship between SARIFA-status and RFS in the subgroup of patients with rectal cancers (*n* = 60). Patients with SARIFA-positive rectal cancers (*n* = 11) had a significantly shorter RFS compared to patients with SARIFA-negative rectal cancers (all pT categories *p* < 0.0001; only pT3/pT4 *p* = 0.0006, respectively). Kaplan-Meier curves for RFS of locally advanced (pT3/pT4) CRCs and the subgroup of patients with rectal cancers can be found in Fig. 3. Kaplan-Meier curves for RFS including all CRCs and rectal cancers regardless of pT category and for cancer-specific survival (CSS) can be found in the supplement (Figure S2 and S3). In univariate Cox regression RFS analysis, higher pT category (pT3/pT4 vs pT1/pT2), higher pN category (pN1/pN2 vs pN0), higher pTNM stage (III/IV vs I/II) and SARIFA-positivity were related to shorter RFS (Table 2). In multivariate Cox regression analysis (Table 2), pT category, pN category, grade of differentiation, PoT and SARIFA-status were included in the model. SARIFA-status remained significantly associated with shorter RFS upon multivariate analysis (p=0.032). SARIFA-positivity as well as low PoT were significantly associated with shorter CSS in univariate analysis (PoT: p=0.023, SARIFA: p<0.001, supplementary Table S2). However, in multivariate analysis neither SARIFA-status nor PoT remained statistically significant (PoT: p=0.091, SARIFA: p=0.051, supplementary Table S3).

**Table 2:** Uni- and Multivariate Cox regression analysis of recurrence-free survival.

	Univariate Cox regression		Multivariate Cox regression*	
	Hazard Ratio (95% CI)	*p-*value	Hazard Ratio (95% CI)	*p-*value
Age>65	0.867 (0.447–1.680)	0.672		
Sex (Female versus male)	0.671 (0.361–1.247)	0.207		
Location (Colon versus rectum)	1.310 (0.700–2.452)	0.398		
pT* (pT1/pT2 versus pT3/pT4)	6.154 (1.897–19.965)	**0.002**	3.951 (0.894–17.464)	0.070
pN* (pN0 versus pN1/pN2)	2.949 (1.574–5.524)	**<0.001**	1.572 (0.690–3.588)	0.282
TNM** (I/II versus III/IV)	2.824 (1.517–5.324)	**0.001**		
Lymphovascular invasion (no versus yes)	1.030 (0.524–2.025)	0.932		
Grade of differentiation* (low versus high)	1.644 (0.835–3.235)	0.150	1.099 (0.430–2.810)	0.844
Proportion of tumour* (low versus high)	0.708 (0.310–1.617)	0.412	1.024 (0.436–2.402)	0.957
SARIFA* (negative versus positive)	3.665 (1.949–6.890)	**<0.001**	2.595 (1.085–6.205)	**0.032**

*p*-values that are statistically significant are highlighted in **bold**.

⁎ Multivariate Cox model was adjusted for pT, pN, grade of differentiation, proportion of tumour.

⁎⁎ Tumour-Node-Metastasis was obtained using TNM, 5th edition (Sobin and Wittekind, 1997, pp 66–69) [22]. CI: confidence interval, pT: depth of invasion, pN: lymph node status, SARIFA: Stroma AReactive Invasion Front Areas.

### Proliferation marker Ki67 and MMR status in relation to SARIFA-status

To evaluate further aspects of tumour biology with respect to SARIFA-status and to better characterize SARIFA-positive CRCs, we investigated the association between SARIFA-status, percentage of Ki67 positive tumour cells and MMR status. There was no association between SARIFA-status and percentage of Ki67 positive tumour cells (*p* = 0.251). SARIFA-status was not associated with MMR status (*p* = 0.565). The results of these immunohistochemical studies are summarized in supplementary Table S4.

## Discussion

The stratification of patients with colorectal cancer (CRC) to identify individual patients at higher risk of recurrent disease remains challenging [4]. To address this pressing clinical need, we validated the prognostic value of our recently established biomarker SARIFA (**S**troma **AR**eactive **I**nvasion **F**ront **A**reas) in an independent external validation cohort of 164 CRC resection specimens. SARIFA-status is assessed using routine Haematoxylin & Eosin (H&E) stained tissue sections from CRC resection specimens. Another known H&E based prognostic biomarker in CRC [9], [10], [11], [12], [13], [14] is the intratumoural stroma content. Whilst SARIFA-status can only be assessed in tissue regions where tumour cells have the opportunity to get into contact with fat cells (especially in the submucosa and subserosa/adventitia), the intratumoural stroma content can be measured in all regions of the tumour including in material from the luminal tumour surface e.g. endoscopic biopsies, see our previous study [14]. Therefore, we investigated the hypothesis that SARIFA-status measured at the invasion front is related to the intratumoural stroma content measured at the endoscopically reachable, luminal tumour surface. Furthermore, we investigated whether SARIFA-status was associated with the percentage of Ki67 positive tumour cells and DNA mismatch repair (MMR) status.

In the current study, SARIFA-status was not associated with MMR status, which is consistent with our own previous reports on CRC as well as gastric cancer (GC) [17,18,19]. SARIFA-status was also not associated with the percentage of Ki67 positive tumour cells which is similar to our findings in GC [17].

We provide first evidence that SARIFA-status might be a prognostic biomarker in patients with rectal cancer. Currently, involvement of the circumferential resection margin (CRM) is one of the key prognostic factors in rectal cancer patients. Considering that many rectal cancers are nowadays CRM-negative due to improved surgical techniques and widespread introduction of neoadjuvant chemoradiotherapy [28], SARIFA-status may be able to substratify patients with CRM-negative rectal cancer. In our view, further studies in larger series of rectal cancers are warranted to investigate this in more detail.

Our current study validated the prognostic value of SARIFA-status in colon cancer patients. Whereas we evaluated H&E stained resection slides manually to determine the SARIFA-status, other investigators using unsupervised deep-learning methodology also identified tumour cells close to adipocytes as a prognostically relevant feature in CRC [[29], [30], [31]], independently supporting our findings. With respect to potential clinical implementation in the near future, it is noteworthy that manual SARIFA-status assessment does not require digital slides or high performance computing power for the analyses, takes on average less than a minute, has shown low interobserver variation [17,18], and hence can be assessed in any pathology laboratory in the World at minimal extra cost (pathologist's time) and no delay in turnaround time.

Whereas our initial SARIFA study only included patients with locally advanced (pT3/pT4) colon cancer [18], the current study also investigated SARIFA-status in early CRCs (pT1/pT2). However, probably not unexpected, only one of 49 (2.0%) CRC showed SARIFA-positivity due to direct contact of tumour cells with submucosal adipose tissue. Our findings could suggest that SARIFA-positivity might be rare in CRCs infiltrating the submucosa. This might simply be related to the amount of fat present in the submucosa (lack of opportunity) or to different underlying biology of tumours infiltrating submucosal fat compared to those infiltrating subserosal fat. Future studies in larger cohorts of early CRCs including malignant polyps are necessary to investigate the potential relevance of SARIFA-status as biomarker in early CRCs.

Interestingly, SARIFA-positive CRCs were characterized by a lower vessel content at the luminal tumour component. Even though there are some studies investigating the relationship between vessel density and outcome in CRC [[32], [33], [34], [35], [36]], further studies are necessary to explore the exact role of vessel density on tumour biology in CRCs, especially with a focus on changes between luminal component and invasion front.

SARIFA-positivity determined at the invasion front was significantly related to low proportion of tumour (PoT) per tumour area measured at the luminal surface. Low PoT is equivalent to high intratumoural stroma, which has been consistently associated with poorer outcomes in CRC [9], [10], [11], [12], [13], [14]. SARIFA-positive CRCs are characterized by a lack of desmoplastic stroma reaction when the tumour cells get into contact with fat cells. Nevertheless, our results suggest that SARIFA-positive tumours are characterised by a higher stroma content at the luminal surface, e.g. opposite to the invasion front. One could speculate that in some CRCs tumour cells benefit from adipocytes as energy providers and by using lipids in numerous signalling pathways, and do not induce a desmoplastic stroma reaction when contacting adipocytes, which, if to say, serve as their ‘partners in crime’ in tumour progression [[37], [38], [39]]. We have shown previously in gastric cancer that SARIFA-positivity is associated with upregulation of fatty acid metabolism in tumour cells [17]. Alexander et al. recently demonstrated that CRCs with a high stroma percentage and low peritumoral inflammation at the invasion front, both features of SARIFA-positive cancers, are characterized by high recurrence rates [40], supporting our hypothesis that SARIFA-positive CRC are characterised by a different tumour microenvironment. If this hypothesis can be confirmed in independent series, this might allow to predict SARIFA-status in pre-treatment/diagnostic endoscopic biopsies – especially considering that also the relative number of vessels measured at the luminal tumour component was also associated with SARIFA-positivity in the current study.

The current study has some limitations. The overall number of CRC patients is relatively small, and especially the relationship between SARIFA-status and prognosis in rectal cancer patients needs to be considered as hypothesis generating result. As only 22 patients received adjuvant therapy, analysis of the relationship between adjuvant therapy, SARIFA-status and patient outcome was not feasible. Additionally, the rates of adjuvant therapy usage are likely to be different in modern cohorts. Surgical techniques, chemotherapy regimens as well as supportive care may also have improved over time, leading to generally better survival nowadays. However, conventional cytotoxic chemotherapy is still standard of care in the adjuvant setting in CRC patients as targeted or immunotherapeutic approaches are generally only considered in the metastatic setting. Moreover, pathologic specimen work up has been improved resulting in a potentially higher lymph node yield in a more recent CRC cohort [[41], [42]]. Another limitation of the study is that only one single representative tumour slide was investigated for SARIFA-status, as described in the methods. However, we do not expect that selection of a representative slide would be different nowadays. Furthermore, we have already demonstrated (at least for a small cohort) that in most SARIFA-positive CRCs the majority of slides show the presence of SARIFA [18]. Immunohistochemical data (MLH1, MSH2, Ki67) was generated using TMAs constructed from regions with highest tumour cell content and not specifically from tumour located next to fat cells. Thus, considering the known heterogeneity of Ki67 staining in different areas in CRC, the Ki67 status might not be representative of the tumour cells that were assessed for the SARIFA-status [[43], [44]].

## Conclusion

In summary, the current study was able to validate the prognostic value of the SARIFA-status in a CRC series which was independent of our initial discovery colon cancer series. The current study provides first evidence that SARIFA-status might be also prognostically relevant in the subgroup of rectal cancer patients. Our results suggest that SARIFA-status in combination with luminal tumour composition may reflect a subset of morphologically (and most likely biologically) distinct CRCs with particular clinical importance. Both parameters can be determined in routine H&E stained sections with low interobserver variability [17],[18]. Consequently, as stromal morphometry (e.g., PoT) alone failed to predict response to adjuvant fluorouracil/folinic acid based chemotherapy in the QUASAR trial [10], it would be interesting to investigate whether a combined score of the characteristics of the tumour microenvironment (PoT, stroma content, vessel density) and SARIFA-status may improve therapy response prediction. Identifying CRC patients at risk, who are likely to benefit from adjuvant therapy, is still a pressing clinical need [45], where assessment of SARIFA-status could be a cost-effective and potent option.

## Author contributions

All authors revised the article critically, contributed to it with reflective improvements, and approved the final version. HG, BM, and NR contributed to the study's conception and design. NW, PQ, AW, WM, HG, and NR contributed to the data acquisition process. BM, HG, JE, BG and NR contributed to the data analysis and interpretation. Finally, BM, HG, BG, and NR wrote the manuscript, and all authors revised the manuscript and approved its final version.

## Data availability

The datasets analysed during the current study are available from the corresponding author upon reasonable request.

## Ethical approval and consent to participate

The study was conducted in compliance with the Declaration of Helsinki. The study was performed in accordance with local ethics regulation, the need for patient consent was waived by the ethics committee.

## CRediT authorship contribution statement

**N.G. Reitsam:** Conceptualization, Data curation, Investigation, Visualization, Writing – original draft, Writing – review & editing. **B. Grosser:** Data curation, Formal analysis, Writing – original draft, Writing – review & editing. **J.S. Enke:** Formal analysis, Writing – review & editing. **W. Mueller:** Data curation. **A. Westwood:** Data curation. **N.P. West:** Data curation, Writing – review & editing. **P. Quirke:** Data curation, Writing – review & editing. **B. Märkl:** Conceptualization, Supervision, Validation, Writing – original draft, Writing – review & editing. **H.I. Grabsch:** Conceptualization, Data curation, Supervision, Validation, Writing – original draft, Writing – review & editing.

## Declaration of competing interest

HG received honoraria from Astra Zeneca and Bristol Myers Squibb not related to the study. BM has received compensations of travel expenses and fees for advisory board activities by Astra Zeneca, Boehringer Ingelheim, MERCK, MSD, BMS, Bayer and Novartis, not related to this study. NW has undertaken paid consultancy for Bristol Myers Squibb, GSK, Astellas and Amgen not related to this study. PQ declares research funding from Roche and honoraria for lectures by Roche, Bayer, Amgen, not related to the study.

## Appendix Supplementary materials

Image, application 1
