# Supplementary material for: Stroma AReactive Invasion Front Areas (SARIFA): a novel histopathologic biomarker in colorectal cancer patients and its association with the luminal tumour proportion
Source: Transl Oncol. 2024 Apr 10;44:101913. doi: 10.1016/j.tranon.2024.101913 (PMC11024380; doi:10.1016/j.tranon.2024.101913)
Supplement: Supplementary file 1 [file mmc1.docx]

*Supplementary Material (Table S1, Table S2, Table S3, Table S4, Figure S1, Figure S2, Figure S3)*

## **Stroma AReactive Invasion Front Areas (SARIFA): a novel histopathologic H&E biomarker in colorectal cancer and its association with luminal tumour proportion**

N.G. Reitsam^1^, B. Grosser^1^, J. S. Enke^2^, W. Mueller^3^, A. Westwood, N.P. West^4^, P. Quirke^4^, B. Märkl^1*^, H. I. Grabsch^4,5*^

## **^*^**H.I. Grabsch and B. Märkl share senior authorship and corresponding authorship.

1 Pathology, Faculty of Medicine, University of Augsburg, Augsburg, Germany.

2 Nuclear Medicine, Faculty of Medicine, University of Augsburg, Augsburg, Germany.

3 Gemeinschaftspraxis Pathologie, Starnberg, Germany

4 Division of Pathology and Data Analytics, Leeds Institute of Medical Research at St James’s University, University of Leeds, Leeds, UK.

5 Department of Pathology, GROW School for Oncology and Reproduction, Maastricht University Medical Center, Maastricht, The Netherlands.

| ^Table S1. REporting recommendations for tumour MARKer prognostic studies (REMARK)^ | |
| --- | --- |
| **^Number (REMARK)^** | **^Reference to Main Manuscript & Comment^** |
| ^1.: study objectives, hypotheses^ | ^Last two paragraphs of the introduction: 1. validation of prognostic relevance of SARIFA-status, 2. and investigation of SARIFA in rectal cancer patients, 3. association between PoT and SARIFA^ |
| ^2./3.: patients: characteristics and treatments^ | ^Material & Methods ('Patients'). 22 patients received adjuvant therapy (no precise treatment regimens available). Patients treated with neoadjuvant therapy were excluded.^ |
| ^4.: specimen characteristics^ | ^Material & Methods ('Patients'). CRC resection specimens, stained with H&E, scanned at 40x^ |
| ^5.: assay methods^ | ^Material & Methods ('SARIFA Assessment'). In particular, assessment of SARIFA-status was performed on one single representative H&E stained tissue section scanned at 40x and reviewed digitally.^ |
| ^6.-9.: study design^ | ^Material & Methods ('Patients'). Retrospective study of consecutive cases without matching or randomization (between January 1990 and December 1995); endpoints: cancer-specific and recurrence-free survival^ |
| ^10./11.: statistical analysis methods^ | ^Material & Methods ('Statistical Analyses'). For verification of assumption of proportionate hazards, we refer to Table S1 and Figure S1.^ |
| ^12./13.: data^ | ^Study flow chart is depicted in Figure 1. All Kaplan-Meier curves are provided with risk tables. Number of events for multivariate Cox Regression model is given in Table S1. Clinicopathological characteristics are provided in Table 1.^ |
| ^14./15.: analysis and presentation^ | ^Relation of SARIFA-status to standard prognostic variables is shown in Table 1. Univariate and multivariate Cox Regression Analysis is provided in Table 2 and S3. As recommended, Kaplan-Meier plots are given in Figure 3, S2, and S3. Confidence intervals as well as p-values are reported.^ |
| ^19./20.: discussion^ | ^Limitations of the current study are discussed in the last paragraph of the discussion. Future implications of our work are also provided. Predictive value of SARIFA alone or in combination with other biomarkers should be assessed. Our findings with regards to higher intratumoural stroma content on the luminal component may enable assessment of SARIFA-status on pretherapeutic biopsies if further studies build upon this. SARIFA-status in rectal cancer as well as pT1/pT2 CRCs should be investigated in larger cohorts.^ |
| ^CRC: colorectal cancer, H&E: haematoxylin and eosin, pT: depth of invasion, PoT: proportion of tumour, SARIFA: Stroma AReactive Invasion Front Areas.^ | |

| Table S2. Parameters of Multivariate Cox Regression Model | | | | |
| --- | --- | --- | --- | --- |
| *Recurrence-Free Survival* | Test of the proportional hazards assumption using Schoenfeld residuals | | | Variance Inflation Factor (VIF) |
| *n= 110, number of events=27* | chisq | df | *p-value* |  |
| pT | 2.677 | 1 | 0.1 | 1.061382 |
| SARIFA | 1.825 | 1 | 0.18 | 1.29577 |
| Grade of, differentiation | 0.195 | 1 | 0.66 | 1.167193 |
| Proportion of tumour | 0.341 | 1 | 0.56 | 1.057896 |
| pN | 0.633 | 1 | 0.43 | 1.166839 |
| *Global* | 4.767 | 5 | 0.45 |  |
|  |  |  |  |  |
| *Cancer-Specific Survival* |  |  |  |  |
| *n=107, number of events=26* | chisq | df | *p-value* |  |
| pT | 0.5969 | 1 | 0.44 | 1.061382 |
| SARIFA | 2.313 | 1 | 0.128 | 1.29577 |
| Grade of differentiation | 2.3293 | 1 | 0.127 | 1.167193 |
| Proportion of tumour | 0.0366 | 1 | 0.848 | 1.057896 |
| pN | 2.8368 | 1 | 0.092 | 1.166839 |
| *Global* | 5.3728 | 5 | 0.372 |  |
| pT: depth of invasion, pN: lymph node status, SARIFA: Stroma AReactive Invasion Front Areas  chisq: chi-square test statistic, df: degrees of freedom; Global: global test for proportional hazards assumption. | | | | |

| Table S3. Uni- and Multivariate Cox Regression Analysis regarding cancer-specific survival | | | |  |
| --- | --- | --- | --- | --- |
|  | Univariate Cox Regression | | Multivariate Cox Regression | |
|  |  |  | adjusted for* | |
|  | Hazard Ratio (95% CI) | *p* value | Hazard Ratio (95% CI) | *p-*value |
| Cancer-specific survival |  |  |  |  |
| Age>65 | 2.249 (0.996-5.078) | 0.051 |  |  |
| Sex (Female vs Male) | 0.469 (0.254-0.870) | **0.016** |  |  |
| Location (Colon vs Rectum) | 1.014 (0.537-1.915) | 0.965 |  |  |
| pT* (pT1/pT2 vs pT3/pT4) | 2.220 (0.984-5.009) | 0.055 | 1.618 (0.522-5.012) | 0.404 |
| pN* (pN0 vs pN1/pN2) | 1.772 (0.958-3.274) | 0.068 | 1.345 (0.587-3.083) | 0.484 |
| TNM** (I/II vs III/IV) | 1.696 (0.918-3.135) | 0.092 |  |  |
| Lymphovascular Invasion (no vs yes) | 1.325 (0.694-2.528) | 0.394 |  |  |
| Grade of Differentiation* (low vs high) | 1.8699 (0.983-3.671) | 0.056 | 1.825 (0.755-4.412) | 0.182 |
| Proportion of Tumour* (low vs high) | 0.404 (0.185-0.881) | **0.023** | 0.501 (0.225-1.116) | 0.091 |
| SARIFA* (negative vs positive) | 3.323 (1.768-6.245) | **<0.001** | 2.428 (0.998.5.907) | 0.051 |
| *p*-values that are statstically significant are higlighted in **bold**. *Multivariate Cox model regarding prognostic value of SARIFA was adjusted for known risk factors: pT, pN, grade of differentiation, proportion of tumour. **Tumour-Node-Metastasis was obtained using TNM, 5th edition (Sobin and Wittekind, 1997, pp 66–69)^19^. CI: confidence interval, pT: depth of invasion, pN: lymph node status, SARIFA: Stroma AReactive Invasion Front Areas | | | | |

| Table S4. Relationship between SARIFA-status, % Ki67 positive tumour cells and mismatch repair status | | | | | |
| --- | --- | --- | --- | --- | --- |
|  | **SARIFA-positive** | | **SARIFA-negative** | |  |
|  | n | % | n | % | *p-value* |
| *Mismatch Repair* (n=161) |  |  |  |  |  |
| proficient | 33 | 89.2 | 106 | 85.5 | 0.565 |
| deficient | 4 | 10.8 | 18 | 14.5 |  |
| *Proliferation (Ki67 expression, n=164)* | |  |  |  |  |
| low/moderate* | 7 | 18.9 | 36 | 28.3 | 0.251 |
| high* | 30 | 81.1 | 91 | 71.7 |  |
| According to the percentage of Ki67 positive tumour cells were categorised into low (<10%)/ moderate (>10% and <25%) vs high (>25%) groups, based on the findings of Melling et al. in colorectal cancer ^20^. | | | | | |

**Figure S1: Schoenfeld Residuals of Multivariate Cox Regression Analysis.**

A. Schoenfeld Residuals over Time for Recurrence-Free Survival; no pattern within time can be observed. Test statistics are provided in Table S2. All p-values are above 0.05.

B. Residuals over Time for Cancer-Specific Survival; no pattern within time can be observed. Test statistics are provided in Table S2. All p-values are above 0.05.

**Figure S2: Kaplan-Meier curves for recurrence-free survival (RFS) stratified by SARIFA-status.** A. Including all pT categories, patients with SARIFA-positive colorectal cancer (n=37) have significantly shorter RFS (HR 3.665, 95% CI: 1.949-6.890, p<0.0001).

B. Patients with SARIFA-positive rectal cancer (n=11) have significantly shorter RFS (HR 7.631, 95% CI: 2.894-20.120, p<0.0001).

HR: hazard ratio, CI: confidence interval. SARIFA: Stroma AReactive Invasion Front Area.

**Figure S2: Kaplan-Meier curves for cancer-specific survival (CSS) stratified by SARIFA-status.**

A. Including all pT categories, patients with SARIFA-positive colorectal cancer (n=35) had significantly shorter CSS (HR 3.323, 95 % CI: 1.768-6.245, p<0.0001).

B. In subgroup analysis, patients with SARIFA-positive rectal cancer (n=10) had significantly shorter CSS (HR 4.033, 95% CI: 1.366-11.910, p=0.0065).

C. In subgroup analysis, patients with locally advanced (pT3/pT4), SARIFA-positive colorectal cancer had significantly shorter CSS (HR 3.031, 95% CI: 1.539-5.968, p=0.00076).

D. In subgroup analysis, patients with locally advanced (pT3/pT4) SARIFA-positive rectal cancer had shorter CSS (HR 4.620, 95% CI: 1.312-16.27, p=0.0092).

HR: hazard ratio, CI: confidence interval. SARIFA: Stroma AReactive Invasion Front Area.
